# Interaction of *Staphylococcus aureus* persister cells with the host when in a persister state and following awakening

**DOI:** 10.1038/srep31342

**Published:** 2016-08-10

**Authors:** Elin G. Mina, Cláudia N. H. Marques

**Affiliations:** 1 Department of Biological Sciences, Binghamton University, Binghamton, NY, 13902, USA; 2 Binghamton Biofilm Research Center (BBRC), Binghamton University, Binghamton, NY, 13902, USA

## Abstract

Persister cells, a tolerant cell sub-population, are commonly associated with chronic and recurrent infections. However, little is known about their ability to actually initiate or establish an infection, become virulent and cause pathogenicity within a host. Here we investigated whether *Staphylococcus aureus* persister cells initiate an infection and are recognized by macrophages, while in a persister cell status, and upon awakening due to exposure to *cis*-2-decenoic acid (*cis*-DA). Our results show that *S. aureus* persister cells are not able to initiate infections in *A. thaliana* and present significantly reduced virulence towards *C. elegans* compared to total populations. In contrast, awakened *S. aureus* persister cells are able to initiate infections in *A. thaliana* and in *C. elegans* albeit, with lower mortality than total population. Furthermore, exposure of *S. aureus* persister cells to *cis*-DA led to a loss of tolerance to ciprofloxacin, and an increase of the bacterial fluorescence to levels found in total population. In addition, macrophage engulfment of persister cells was significantly lower than engulfment of total population, both before and following awakening. Overall our findings indicate that upon awakening of a persister population the cells regain their ability to infect hosts despite the absence of an increased immune response.

Persister cells, firstly described by Bigger in 1944, are a subpopulation of antimicrobial tolerant cells^1^ and constitute less than 1% of the total population^2^. Upon removal of antimicrobial therapy, the surviving persister cells regrow to a new genetically diverse population identical to the original population^3^, with an identical percent of tolerant cells as the one observed in the original culture^2^.

Persister cell formation has been attributed to inoculum conditions, culture growth stage, antibiotics used, and induction of cell stasis due to the stringent response and increase of polyP compounds^4^,^5^,^6^. Persister cells can form upon induction of a SOS response due to an inhibition of DNA repair by ciprofloxacin which leads to DNA damage^7^,^8^,^9^,^10^. The mechanism of ciprofloxacin-induced persister formation was found, in *Escherichia coli*, to be partially due to the expression of the TisB toxin, which is involved in the decrease of ATP levels and the stop of the proton motive force^9^,^10^. Toxin-antitoxin (TA) systems-where a stable toxin inhibits the cellular functions, and an unstable antitoxin neutralizes the toxin-have also been demonstrated to be involved in persister cell formation^10^,^11^,^12^. One such TA systems is MqsR/MqsA, where the MqsR toxin is highly expressed resulting in an increase of *E. coli* persister cell formation^13^. The MqsR toxin is dependent on Hha (a toxin-antitoxin system) and CspD (a stress-induced cold shock protein and DNA replication inhibitor) that results in the formation of persister cells^10^,^13^.

Several approaches have been attempted to revert the tolerant state of persister cells, including the use of a synthetic brominated furanone, (*Z*)-4-bromo-5-(bromomethylene)-3-methylfuran-2(5*H*)-one (BF8) that inhibits *lasB* in *Pseudomonas aeruginosa* PAO1 and leads to persister eradication^14^. Exposure to 3-[4-(4-methoxyphenyl)piperazin-1-yl]piperidin-4-yl biphenyl-4-carboxylate (C10) reverts persister cells to an antibiotic susceptible state, without affecting the cells’ susceptibility to antimicrobials^15^. Killing of *E. coli* persister cells also occurred upon exposure to cationic membrane-penetrating peptides (arginine and tryptophan)^16^. The use of carbon sources (glucose and mannitol), as metabolic stimuli has resulted in an overall increase in *Staphylococcus aureus* and *E. coli* persister cells’ respiration and central metabolism, increasing the efficacy of aminoglycosides^17^. The antibiotic acyldepsipeptide (ADEP-4) was shown to activate the ClpP protease leading to cell degradation and resulting in eradication of persister cells^18^,^19^. More recently, mitomycin C was shown to eradicate persister cells of *E. coli*, *S. aureus*, *P. aeruginosa* and *Borrelia burgdorferi*^20^,^21^. The reversion of a persister cell state has also been achieved with the fatty acid signaling molecule *cis*-2-decenoic acid (*cis*-DA), where *P. aeruginosa* and *E. coli* increased their metabolic status without increasing their active growth, and upon exposure to antibiotics a significant decrease of the cell numbers was achieved to the point of eradication^22^.

Although it is widely accepted that persister cells play a role in chronic infections and infection recurrence^5^,^19^,^23^, to our best knowledge, the virulence of persister cells and their ability to infect and kill a host has yet to be demonstrated. Motivated by this knowledge gap, we investigated whether *Staphylococcus aureus* persister cells cause virulence in the *Arabidopsis thaliana* and *Caenorhabditis elegans* host models, and whether they are able to be ingested by THP-1 macrophages. In addition, we evaluated whether exposure to *cis*-DA can lead to *S. aureus* persister cell awakening and alteration of *S. aureus* virulence and pathogenicity within a host.

## Results

### Isolation and confirmation of persister cell state

**Figure 1:**
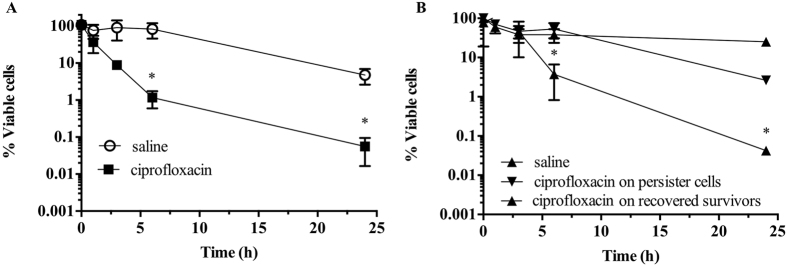
Isolation of persister cells from *S. aureus* planktonic populations. Stationary-phase planktonic cultures were exposed to saline or ciprofloxacin in saline for a period of 24 h (**A**). Cell viability was determined at 0, 1, 3, 6 and 24 h. To confirm that only persister cells were present, cultures were washed and isolated persister cells were exposed to saline or ciprofloxacin in saline for 24 h (**B**). Persister cells were also re-grown in LB medium and recovered survivors were re-challenged with ciprofloxacin for a period of 24 h (**B**). The averages of data from 3 experiments with 2 replicates per experiment are shown. Error bars indicate standard deviations (*P < 0.001- significantly different from cells exposed to saline alone, as indicated by one-way ANOVA).

Our laboratory has previously isolated persister cell populations from biofilm and planktonic cultures of *E. coli* and *P. aeruginosa* using the SOS response^22^. Similarly, in this work, persister cell populations were isolated from stationary phase planktonic cultures of *S. aureus* using ciprofloxacin, by inducing the SOS response^2^,^7^,^22^,^24^,^25^. Previously it has been demonstrated that ciprofloxacin lysis bacterial cells of *E. coli*, *P. aeruginosa* and *Enterobacter cloacae*^26^,^27^,^28^. We observed a typical biphasic killing, with a high killing slope with a significant decrease (P < 0.001) of cell viability within the initial 6 h of treatment, followed by a minor killing slope (Fig. 1A). The final recovery of persister cells, was <1%, an observation consistent with previous findings^19^. The persister cell state was confirmed in two ways: by assessing the viability of persister cells upon subsequent exposure to antimicrobials^22^,^29^, and by re-growing and re-isolating persister cells^29^ (Fig. 1B). *S. aureus* persister cells were washed and subsequently exposed to saline or ciprofloxacin in saline for an additional 24 h without the presence of any further killing (Fig. 1B). Following the washing, persister cells were also re-grown in 100% LB, after which exposure to ciprofloxacin ensued with an identical killing and percent of persister cells as the one observed during the initial persister isolation (Fig. 1A,B). Thus, there was no significant difference in viability (P > 0.1) observed between persister subpopulations when exposed to saline or ciprofloxacin. Upon re-growth, an identical biphasic killing curve was observed which confirmed persister state of these population.

### *Arabidopsis thaliana* is not affected by persister cells

**Figure 2:**
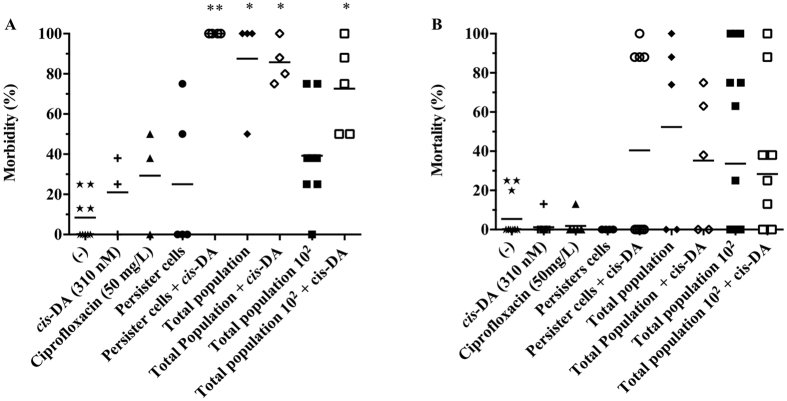
Effect of persister cells on *A. thaliana* morbidity and mortality. *A. thaliana* plants were infected with *S. aureus* persister cells, total population (at identical concentration as persister cells), and total population 10^2^, in the presence and absence of *cis*-DA (310 nM). Controls consisted of ½ MS salts (−), *cis*-DA (310 nM) alone and ciprofloxacin (50 mg/L). *A. thaliana* morbidity 5 days post infection (**A**). *A. thaliana* mortality 7 days post infection (**B**). Results shown consist of a minimum of 5 experiments, using 8 plants per experiment. Lines indicate average. *Significant infection compared to (−), and *cis*-DA (P < 0.001); **Significant infection compared to: (−), *cis*-DA, and equivalent treatment without *cis*-DA (P < 0.01); as indicated by one-way ANOVA followed by Tukey’s multicomparison test.

*A. thaliana* are small flowering plants widely used as model organisms to assess bacterial virulence and pathogenicity, as their innate immune system shares similarities to the mammalian innate immune system^30^. This model has previously been used as a model for *S. aureus* infections, where it was shown that *agrB* and *sarA* virulence regulators were important in plant pathogenesis^31^. These findings led us to investigate whether persister cells are able to establish an infection and induce morbidity and mortality in *A. thaliana*. Plants were infected with persister cells (10^4^ CFU/mL), total population at an identical concentration to persister cells (10^4^ CFU/mL) - called henceforth total population, and total population at its standard concentration when in stationary phase containing active cells and ≤1% persister cells (10^6^ CFU/mL) - called henceforth total population 10^2^. At day 5 of infection, a ≤30% *A. thaliana* morbidity was recorded in plants infected with persister cells, ciprofloxacin alone (control), and medium alone (control) (Fig. 2A). Infections with total population and total population 10^2^ led to a >80% and 40% morbidity, respectively. Similarly to morbidity, by day 7 the plant mortality (Fig. 2B) was identical in infections with persister cells and controls <20%, whilst mortality was recorded to be >30% in total population 10^2^ and >40% in total population (Fig. 2B). An identical *A. thaliana* morbidity and mortality was recorded on infections with persister cells in the presence or absence of ciprofloxacin (not shown). Our findings suggest that *S. aureus* persister cells are not able to infect or cause pathogenicity in the plant model.

### *S. aureus* persister cells have a low virulence against *C. elegans*

**Figure 3:**
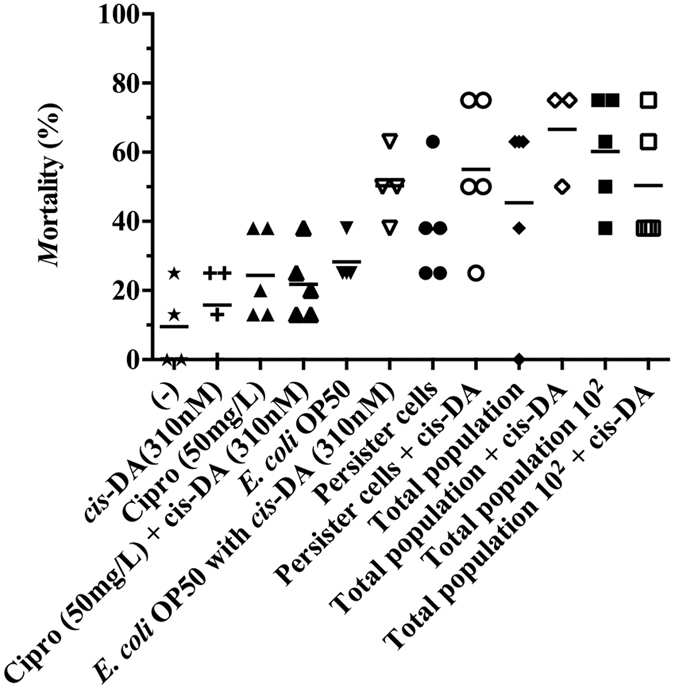
Effect of persister cells on *C. elegans* mortality when using the slow killing assay. *C. elegans* nematodes were infected with *S. aureus* persister cells, total population (at identical concentration as persister cells), and total population 10^2^, in the presence and absence of *cis*-DA. *C. elegans* mortality was assessed daily for a period of 15 d. Overall mortality was assessed at 15 d. Results shown consist of a minimum of 4 experiments, using 8 nematodes per experiment.

**Figure 4:**
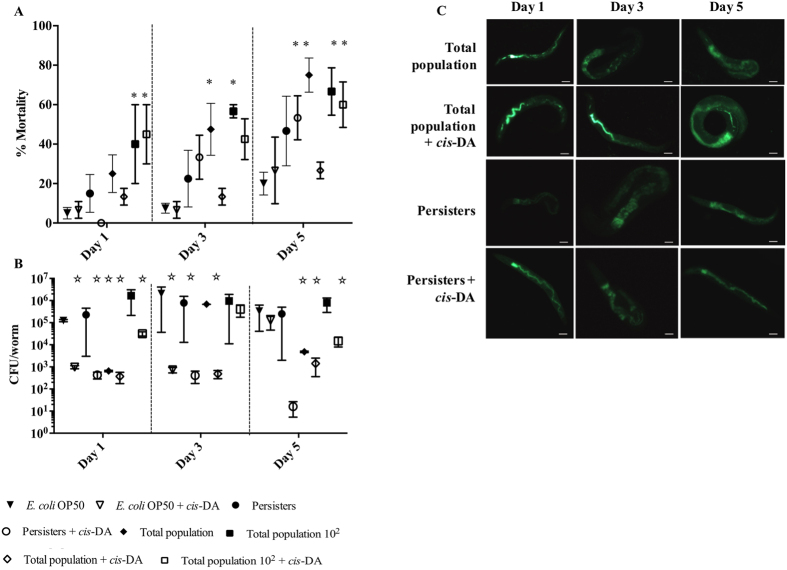
Effect of persister cells on *C. elegans* mortality when using the fast killing assay. *C. elegans* nematodes were infected with *S. aureus* persister cells, total population (at identical concentration as persister cells), and total population 10^2^, in the presence and absence of *cis*-DA (310 nM). *C. elegans* infection was followed for a period of 5 days. *C. elegans* mortality was scored at 1, 3 and 5 d (**A**). *S. aureus* CFU per worm were determined at 1, 3 and 5 d (**B**). Bacterial fluorescence and distribution within the nematode was also assessed (**C**). Results shown consist of a minimum of 4 experiments, using 10 nematodes per experiment. Bar corresponds to 100 μm. Error bars indicate standard error of the mean. *Significant death compared to *E. coli* (P < 0.001); Significant lower CFU/worm compared to *E. coli* (P < 0.001); as indicated by one-way ANOVA followed by Tukey’s multicomparison test.

*C. elegans,* small transparent multicellular eukaryotic organisms that feed on *E. coli* OP50, are used as model organisms to investigate virulence mechanisms and immune response towards microorganisms^32^. In the absence of morbidity and mortality in the plant model, we investigated whether *S. aureus* persister cells are pathogenic towards nematodes, as *S. aureus* is considered to be an animal pathogen^31^. To achieve this, we made use of the slow killing (Fig. 3), and the fast killing (Fig. 4) assays. In the slow killing assay (Fig. 3), *C. elegans* nematodes were infected with persister cells, total population, and total population 10^2^, for a period of 15 d in liquid media. At day 15, infections initiated with persister cells resulted in mortality similar to controls and significantly lower than infections initiated with total populations (Fig. 3A). Mortality upon infection with persister cells was identical in the presence and absence of ciprofloxacin (not shown). In order to ensure that the variation of nematode mortality was not due to an increase of the bacterial load, viable counts were performed, at day 15. No significant difference (P > 0.05) in CFU/nematode was observed between the various infections indicating that differences of *C. elegans* mortality was not related to an increase of the bacterial load.

Mortality of *C. elegans* in fast killing assays (Fig. 4) was identical in controls and infections with persister cells, at day 1 however, mortality in infections with persister cells was higher than controls at days 3 and 5 (Fig. 4A), albeit not being significant. Nematode mortality in infections initiated with total population 10^2^, remained relatively constant (50–60%) over the 5-day period, while in infections initiated with total population the mortality significantly increased overtime (P < 0.01) reaching 80% at day 5 (Fig. 4A). When monitoring bacterial viability, we found that the CFU/nematode remained constant overtime in infections with persister cells, total population 10^2^ and controls (*E. coli* OP50), while the total population, was initially reduced and subsequently increased to the levels of the other infections (Fig. 4B). Due to the variation on bacterial viability, we decided to evaluate the expression of green fluorescence of *S. aureus* RN6390 during the fast killing infections (Table 1). During infection with persister cells, the fluorescence was initially 1 Log lower than infections with total population however, at day 5 persister cells’ fluorescence reached fluorescent levels of the total population. Our findings indicate that persister cells potentially remain in the persister state within the first 3 days of infections, supported by the fact that the bacterial load and fluorescence is identical at days 1 and 3, and subsequently revert their status to an active state similar to the total population 10^2^ by day 5. Therefore, persister cells once ingested by *C. elegans* behave as active cells resulting in the increase of nematode mortality over the 5-day period relative to *E. coli* alone.

### Macrophages engulf *S. aureus* persister cells but at a lower rate than total bacterial population

**Figure 5:**
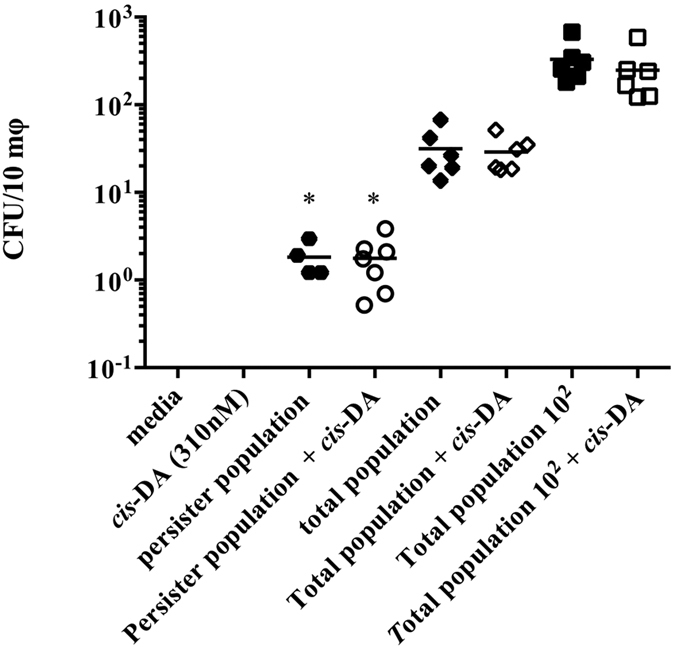
Macrophage uptake of *S. aureus* populations. THP-1 monocytes (5 × 10^5^) were allowed to differentiate for a period of 72 h, after which, they were exposed to *S. aureus* persister cells, total population (at identical concentration as persister cells), and total population 10^2^, in the presence and absence of *cis*-DA. Following 1.5 h, intracellular *S. aureus* CFUs were evaluated. Results shown consist of a minimum of 4 experiments. Lines indicate average. (*P < 0.001- significant lower engulfment compared to total population and total population 10^2^, as indicated by one-way ANOVA).

Macrophages (mΦ) act as an innate immune response and secrete cytokines which then attract other immune cells to the site of infection^33^. The bacterial engulfment is established by pattern recognition receptors (PRRs)^34^, which detect the presence of pathogen-associated molecular patterns (PAMPs) found on microorganisms^35^. Once a macrophage recognizes a pathogen, engulfs it by forming phagosomes, followed by its elimination^36^,^37^. The evidence that infections initialized with persister cells are not significantly different from the negative controls when infecting *A. thaliana* (Fig. 2) and *C. elegans* (Figs 3 and 4) led us to investigate whether macrophages are able to recognize and engulf persister cells. THP-1 monocytes were differentiated with PMA for a period of 3 d and subsequently exposed to persister cells, total population, total population 10^2^, and medium alone for 1.5 h (Fig. 5). We observed that persister cells were engulfed at a rate of 2 CFU per 10 mΦ, which was significantly (P < 0.001) less when compared to total population and total population 10^2^. Total population was engulfed at a rate 10x higher than persister cells and 10X lower than total population 10^2^. The difference in engulfment could possibly be due to the differences within the cell wall of dormant cells compared to active cells^38^.

### Effect of *cis*-2-decenoic acid (*cis*-DA) on *S. aureus* bacterial growth

Bacterial cells make use of several signaling systems. In the last 20 years, a sub-class of *cis*-2-unsaturated fatty acids has been characterized and found to be involved in intra- and inter-species bacterial signaling, and to be involved in bacterial virulence, biofilm formation, biofilm dispersion, stress tolerance, motility, tolerance to antimicrobials, biofilm dispersion and persister cell awakening^39^. One such molecule is *cis*-2-decenoic acid (*cis*-DA) which, our laboratory has demonstrated to increase the metabolic activity of *Pseudomonas aeruginosa* and *Escherichia coli* persister cells, in both planktonic and biofilm derived populations^22^. This evidence has led us to investigate whether *cis*-DA also awakens *S. aureus* persister cells and changes their virulence and pathogenicity. Efficacy of *cis*-DA in awakening *S. aureus* persister cells has, to our knowledge, never been demonstrated. However, the presence of *cis*-DA together with antimicrobials has been found to reduce the viability of *S. aureus* biofilms, eradicate mixed species biofilms and remove pre-formed biofilms of *B. subtilis*, *S. enterica*, *S. aureus* and *E. coli* from surfaces^40^,^41^,^42^. Therefore, we decided to evaluate the effect of *cis*-DA on *S. aureus* persister cells. We observed that *cis*-DA does not inhibit *S. aureus* bacterial growth at concentrations ranging from 2 nM to 0.1 μM (Fig. S1). We also assessed the growth of *S. aureus* in EPRI liquid medium, using either *cis*-DA or peptone as the sole carbon source (Fig. S2), where *cis*-DA was provided at concentrations ranging from 100 nM to 1000 nM, while peptone was used at concentrations ranging from 0.001% to 0.1%. Using peptone as a sole carbon source, growth was observed for all concentrations tested (Fig. S2A). However, no growth was detected in the presence of *cis*-DA regardless of its concentration (Fig. S2B). These findings indicate that *cis*-DA does not inhibit the growth of *S. aureus* and is not utilized as a carbon source.

### Eradication of *S. aureus* persister cells when exposed to *cis*-DA in combination with ciprofloxacin

**Figure 6:**
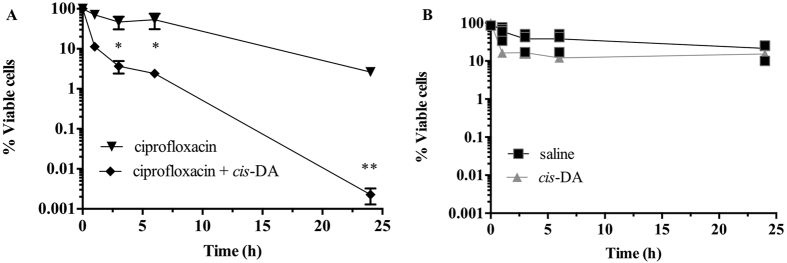
Tolerance of persister cells to ciprofloxacin is reduced in the presence of *cis*-DA. Persister cell sub-populations isolated from *S. aureus* were exposed to ciprofloxacin and ciprofloxacin with *cis*-DA to assess whether they would lose their tolerance upon bacterial awakening (**A**). Persister cells were also exposed to saline and *cis*-DA (310 nM) in saline for a period of 24 h (**B**). The averages of data from 3 experiments with 2 replicates per experiment are shown in A, while individual data are shown in B. Error bars indicate standard deviations (*P < 0.01; **P < 0.001- significantly different from cells exposed to saline alone, as indicated by one-way ANOVA).

Recently, our laboratory has shown that as a result of awakening persister cell populations, exposure of *E. coli* and *P. aeruginosa* persister cells to *cis*-DA previous to- and co-current with- antimicrobials leads to a significant reduction in persister viability, to the point of eradication^22^. To evaluate whether this occurs in *S. aureus*, persister cells isolated from planktonic cell populations were exposed to ciprofloxacin (50 μg/mL) in the presence and absence of *cis*-DA (310 nM) for a period of 24 h. Overall, a significant (P < 0.001) reduction in cell viability of 4.5-Log, to the point of eradication, was observed in the presence of *cis*-DA, whilst in its absence a 1-Log reduction was observed (Fig. 6A). The further 1 Log reduction of the viability of persister cells in the presence of ciprofloxacin was probably due to the stochastic awakening of the persister population^12^. Exposure of persister cells alone resulted in an absence of a statistically significant (P > 0.1) reduction of cell viability when exposing persister cells to saline or *cis*-DA (310 nM) alone (Fig. 6B). These findings indicate that similarity to *E. coli* and *P. aeruginosa*^22^, in *S. aureus, cis*-DA does not inhibit growth and is not used as a carbon source. It does however, contribute to a reduction of the persister cell population, possibly by altering their metabolic status.

### *cis*-DA increases the virulence of *S. aureus* persister cells

Production of *cis*-DA in *P. aeruginosa* is due to the gene *DspI* (PA14_54640/PA0745) which is responsible for catalyzing the formation of α,β-unsaturated fatty acids^43^, and has been demonstrated to increase the virulence of *P. aeruginosa* towards *C. elegans*^44^. Upon finding that the presence of *cis*-DA reverts the tolerant state of *S. aureus* persister cells (Fig. 6), we evaluated whether persister cell’s virulence would be influenced by *cis*-DA. To achieve this, we used two different models, *A. thaliana* (Fig. 2) and *C. elegans* (Figs 3 and 4), as it was previously demonstrated that *sarA* and *agrB* are involved in *S. aureus* virulence in both animals and plants^45^. Contrary to infection with *S. aureus* persister cells alone that led to a low morbidity and an absence of mortality in *A. thaliana* (Fig. 2), the presence of *cis*-DA in infections with *S. aureus* persister cells led to a 100% morbidity by day 5, a significant increase compared to negative controls and the absence of *cis*-DA (p < 0.01) (Fig. 2A). Moreover, the increase of morbidity in the presence of *cis*-DA was also observed in plants infected with the total population 10^2^, where morbidity increased significantly (P < 0.01) from 40% to 70% in the presence of *cis*-DA. In total population, *cis*-DA did not have an effect in morbidity compared to the absence of *cis*-DA. Although, a higher mortality was observed in infections with persister cells in the presence of *cis*-DA, they were not statistically different (P > 0.05). Overall, no difference in mortality was observed for total population 10^2^ and total population in the presence of *cis*-DA (Fig. 2B).

As it is known that *S. aureus* is an animal pathogen, we decided to evaluate its pathogenicity in *C. elegans*. In the slow killing assay (Fig. 3), the presence of *cis*-DA led to an increase of *C. elegans* mortality to 58% from 40% in persister cells infections, and a mortality of 70% from 50% in infections with total populations (Fig. 3). The mortality increase was however, not statistically significant (P > 0.05). No change in mortality was observed in infections with total population 10^2^ (Fig. 3). The change in mortality observed in the presence of *cis*-DA was not due to a change in the bacterial load, as no statistical significant difference (P > 0.05) in CFU/nematode between infections was observed. In the fast killing assay (Fig. 4), the presence of *cis*-DA led to a reduction of the CFU/nematode in all infections and in controls (Fig. 4B). A lower *C. elegans* mortality was observed at day 1, in persister cell infections in the presence of *cis*-DA (P < 0.01), compared to persister cells alone (Fig. 4A). However, on subsequent days, there was an increase in percent mortality in persister cells in the presence of *cis*-DA compared to persister cells alone (Fig. 4A). This is consistent with the significant increase (P < 0.01) of fluorescence in *S. aureus* observed at days 1 and 3 of infection with persister cells in the presence of *cis*-DA, compared to persister cells alone (Table 1). In infections with total population, nematode mortality was significantly reduced throughout the experiment, compared to total population alone (Fig. 4A), corroborating the decrease in the bacterial fluorescence (Table 1). No difference in nematode mortality was observed in the presence or absence of *cis*-DA in infections with total population 10^2^. Our results indicate that upon awakening of persister cells, *S. aureus* increase their virulence towards *A. thaliana* and *C. elegans*.

### *cis*-DA does not influence the bacterial uptake by macrophages

The increased virulence, by awakened *S. aureus* persister cells, towards *A. thaliana* (Fig. 2) and *C. elegans* (Figs 3 and 4), led us to investigate whether these changes would result in an increase of macrophage activation together with an increase of engulfment of the bacterial cells. Following exposure of macrophages for a period of 1.5 h to persister, total and total 10^2^ populations in the presence of *cis*-DA, we observed no difference in the bacterial engulfment, compared to the absence of *cis*-DA (Fig. 5). Previously, it has been suggested that the presence of *cis*-DA does not lead to an activation of bacterial division and growth, but only to cell awakening and increase of the cells’ metabolic activity^22^. While, we hypothesized that persister cells, once exposed to *cis*-DA, would behave similar to normal populations resulting in the same engulfment rate, we observed the opposite. These results could possibly be due to the fact that albeit being awakened, within 1.5 h the bacterial cell wall is not changed and therefore, the bacterial PAMPS in persister cells might not be recognized by the macrophage’s PRR.

## Discussion

Persister cells are commonly thought to be responsible for infection recurrence, however, little is known of their ability to initiate/cause infections once all the active population is removed either by the immune system or killed by the antimicrobials. Recently, evidence showed that in the presence of human granulocyte macrophage-colony stimulating factor (GM-CSF), *P. aeruginosa* persister cell viability is not altered^46^. However, its presence leads to an up-regulation of gene expression and sensitizes the bacteria to several antibiotics^46^. Our study focuses on the ability of *S. aureus* persister cells to initiate an infection, maintain an infection and become engulfed by macrophages while in a persister state and upon being reverted into a metabolically active (awake) state resulting from the exposure to *cis*-2-decenoic acid (*cis*-DA).

Overall *S. aureus* persister cells are engulfed at a lower rate by macrophages, and show a significant lower virulence compared to the normal population as they are unable to infect and kill *A. thaliana* (Fig. 2) or kill *C. elegans* in the slow killing assay (Fig. 3). However, when evaluating persister cell virulence using the fast killing assay, *C. elegans* mortality increased slightly throughout persister cell infections when compared to controls (Fig. 4A). The increase of mortality was concurrent with an increase of bacterial relative fluorescence and an absence of a change in CFU/nematode.

These results led us to hypothesize that once inside a host, persister cells awake and become more virulent and pathogenic. To confirm this, we made use of the signaling molecule *cis*-DA, originally isolated from *P. aeruginosa*^47^ and previously found to awaken persister cells of *P. aeruginosa* and *E.coli* by increasing their respiratory activity, changing the gene expression and leading to the cell’s reduction in viability when used in conjunction with antimicrobials^22^. Likewise, we found that *cis*-DA is not used as a carbon source (Fig. S2), awakens *S. aureus* persister cells, and when used in conjunction with antimicrobials, persister cells’ viability is significantly reduced (Fig. 6). In addition, the presence of *cis*-DA led to an increase of morbidity and mortality of *A. thaliana* (Fig. 2), and of *C. elegans* mortality (Figs 3 and 4). The changes in mortality occurred in the absence of an increase of bacterial cell numbers. The increased mortality and morbidity was not observed in infections with total population 10^2^.

Taken together, our studies show that persister cells are non-virulent for plants (Fig. 2), have a lower virulence towards nematodes (Figs 3 and 4) compared to the total populations and cannot be engulfed by macrophages (Fig. 5). Moreover, awakening of persister cells rends them more virulent without leading to a higher engulfment by macrophages. Our results challenge the hypothesized concept that macrophages are able to remove persister cells, whether in their dormant state or following awakening, and whether they are able to be cleared from a host infection.

## Materials and Methods

### Bacterial strains and growth conditions

Throughout this study *Staphylococcus aureus* ATCC 6538 and *S. aureus* RN6390 *gfp::tet*, constitutively expressing green fluorescent were used^48^,^49^. All overnight cultures were grown on Lennox media (LB, Becton, Dickinson, Sparks, MD) in Erlenmeyer flasks at 37 °C with agitation (220 rpm), unless indicated otherwise.

### Persister cell isolation

*S. aureus* persister cells were isolated from planktonic populations by making use of the activation of the SOS response in the presence of ciprofloxacin, as previously described^7^,^22^,^25^,^29^,^50^,^51^. Briefly, overnight cultures were washed twice with saline, pelleted (16,000 × *g*, for 5 m at 4 °C) and resuspended at a final OD_600_ of 0.8, in either saline or ciprofloxacin (50 μg/ml) in saline. Cultures were subsequently incubated at 37 °C with agitation (220 rpm) for a period of 24 h. Viability was determined at 0, 1, 3, 6 and 24 h. At each time point, 1 mL samples were taken, serially diluted and drop plated onto plate count agar (PCA) plates with 1% MgCl_2_ · 7H_2_O, to inactivate ciprofloxacin^22^. Viability was determined following 24 h incubation at 37 °C.

### Confirmation and maintenance of the persister cell state

Persister cells were isolated from *S. aureus* cultures as described above. Confirmation of the persister cell state was performed as previously described^22^. Briefly, persister cells were exposed to saline or ciprofloxacin in saline and survivor cells were rechallenged with ciprofloxacin for a period of 24 h, at 37 °C. Cell viability was assessed at 0, 1, 3, 6 and 24 h as described above. Persister state was indicated by the maintenance of stable cell viability. When determining the effect of *cis*-2-decenoic acid (310 nM) on persister cell maintenance, persister cells were exposed to *cis*-DA in saline or saline alone for a period of 24 h, at 37 °C. Cell viability was determined at 0, 1, 3, 6 and 24 h as described above. Loss of the persister state was indicated by the loss (decrease) of cell viability.

### Killing efficacy assays

Killing efficacy assays were performed as described before^22^. Briefly, persister cells were exposed to ciprofloxacin alone and ciprofloxacin in combination with the fatty acid signaling molecule *cis*-DA. *S. aureus* persister cells were pelleted, resuspended in 50 mL of saline, and aliquots of 6 mL were subjected to one of the following treatments: saline, *cis*-DA (310 nM) in saline, ciprofloxacin in saline, or ciprofloxacin with *cis*-DA (310 nM) in saline. Cultures were incubated at 37 °C with shaking (220 rpm) for 24 h. Cell viability was determined at 0, 1, 3, 5, and 24 h as described above.

### Planktonic growth curves using *cis*-DA as a carbon source

Planktonic growth curves were performed for *S. aureus* using peptone or *cis*-DA as carbon sources. Overnight cultures were washed twice with saline (16000 × *g* for 5 m, at 4 °C), and subsequently diluted to 1% in minimal EPRI medium (Electric Power Research Institute)^43^,^47^ supplemented with 100 nM, 300 nM or 1000 nM of *cis*-DA, or with 0.001%, 0.01% or 0.1% of peptone. Cultures were grown at 37 °C, in 96-well microtiter plates, with OD_595_ measurements taken every 60 m (DTX880 multimode detector, Beckman Coulter, CA).

### MIC determination

MICs of *S. aureus* to ciprofloxacin were determined in 100% LB using standard methods^52^.

### *Arabidopsis thaliana* infections

The virulence of *S. aureus* persister cells and the tracking of bacterial cell proliferation in *planta* was evaluated with the *A. thaliana* model^45^,^53^. *A. thaliana* seeds were sterilized with 95% ethanol and 2.5% Sodium hypochlorite, seeded onto ½ Murashige and Skoog Basal media (MS) agar (1.1 g/l MS salts, 0.8% agar, 1.5% sucrose) and synchronized in the dark, at 4 °C for 72 h. Subsequently, seeds were allowed to germinate for 12 d, at 25 °C, in a 16 h light/8 h dark cycle. Each plant was then transferred to a well containing 1.5 ml of ½ MS (1.1 g/l Murashige and Skoog Basal media). Following 48 h, plants were infected with *S. aureus* persister cells (10^4^ CFU/mL), *S. aureus* total population at identical concentration of persister cells, and *S. aureus* total population 10^2^ (10^6^ CFU/mL). Infections were performed in the presence and absence of *cis*-DA (310 nM). Controls consisted of *cis*-DA (310 nM) alone and MS alone. Plant infection and death was monitored for 14 d and recorded based on plant wilting, discoloration and necrosis^45^. Infections were performed at least in triplicate with 8 biological replicates per experiment.

### *Caenorhabditis elegans* infections

The ability of *S. aureus* persister cells to infect and cause pathogenicity on *C. elegans* was evaluated via 2 different methods: slow killing in liquid media, and fast killing in solid media^45^,^54^. ***Slow killing assay*** - Previous to infection, L4 molt stage *glp-4*(*bn2*) nematodes were synchronized. Eggs were harvested following exposure of nematodes to 3.75% hypochlorite and 1N sodium hydroxide and transferred onto nematode growth media agar (NGM) pre-seeded with *E. coli* OP50 (food source)^55^. Upon 2 day incubation at 22 °C nematodes were washed 3 times (750 × *g*, for 5 m at 22 °C) with 500 μL of M9 buffer (0.3% monobasic potassium phosphate, 0.5% sodium chloride, 0.6% sodium phosphate dibasic, and 1M magnesium sulfate) and individual nematodes were transferred into individual wells of a 96 well plate, that contained liquid media (97% M9 buffer, 2.5% EPRI and 1% cholesterol). Following 24 h, nematodes were infected with *S. aureus* persister cells (10^4^ CFU/mL), *S. aureus* total population at identical concentration of persister cells, and *S. aureus* total population 10^2^ (10^6^ CFU/mL), in presence and absence of *cis*-DA (310 nM). Controls consisted of medium alone, *cis*-DA (310 nM) alone, and *E. coli* OP50 in presence and absence of *cis*-DA. Mortality was recorded daily for a period of 15 days; nematodes that did not display movement and did not respond to the touch of the microtiter plates were considered dead. Bacterial load present within the nematode was evaluated daily by determining bacterial fluorescence every 12 h (Beckman 680X). To determine bacterial infection load at day 15, nematodes in each condition were pooled, washed 3 times with M9 buffer (750 × *g*, for 5 m at 22 °C) and resuspended in 150 μL of M9 buffer with 1% Triton X-100 to lyse the nematodes. Following 10 m at 22 °C, samples were homogenized with a pestle motor mixer (Argos Technologies), serially diluted and drop plated onto plate count agar (PCA) plates. Viability was determined following 24 h incubation, at 37 °C. Infections were performed in triplicate with 8 replicates per experiment^55^. **Fast killing assay** – Nematodes were synchronized as described above. Following the 2 day incubation, nematodes were washed (750 × *g*, for 5 m at 22 °C) with M9 buffer and5 nematodes were transferred onto each well of a 12 well plate containing NGM agar (NGM, with 1.5% noble agar and without peptone) in the absence or presence of *cis*-DA (310 nM). Agar in each well was pre-seeded with *S. aureus* persister cells (10^4^ CFU/mL), *S. aureus* total population at identical concentration of persister cells, and *S. aureus* total population 10^2^ (10^6^ CFU/mL). Controls consisted of medium alone, *cis*-DA (310 nM) alone, and *E. coli* OP50. Nematode mortality and bacterial load was monitored for a period of 5 d and samples were taken at 1, 3 and 5 d. Bacterial location and load was assessed using cell viability and microscopy. When assessing bacterial CFU, nematodes were transferred from each well onto a microfuge tube containing 200 μL of M9 buffer and subsequently washed 3X with M9 buffer. Samples were processed as described above. When assessing bacterial fluorescence, individual nematodes were placed onto mounting oil and then visualized using an Epifluorescence microscope (Olympus BX46). Infections were performed in quadruplicate with 2 replicates per experiment^56^,^57^.

### Determination of macrophage recognition of *S. aureus* persister cells

To establish whether macrophages engulf persister cells we made use to THP-1 cells. THP-1 cells were cultured in a loose suspension of RPMI 1640 medium supplement with 10% Fetal Bovine Serum (Corning), and the media was changed every 2–3 d. Monocytes at a concentration of 5 × 10^5^ cells per mL were differentiated into macrophages with 100 nM phorbol 12-myristate 13-acetate (PMA, Sigma Chemical Co.) for 3 days at 37 °C, 5% CO_2_. Macrophages were subsequently infected with *S. aureus* persister cells standardized to 10^4^ CFU/mL, *S. aureus* total cells at identical concentration of persister cells (total population), and *S. aureus* total population10^2^ (10^6^ CFU/mL), in presence and absence of *cis*-DA (310 nM), for a period of 90 m. Quantitation of bacterial cells engulfed was assessed following the removal of extracellular bacterial cells with gentamycin (40 mg/L) for a period of 1 h^52^. THP-1 cells were subsequently trypsinized and collected onto microfuge tubes. THP-1 cells were then exposed to Triton X-10 for 45 m to lyse the macrophages and release the intracellular bacteria onto the liquid. Cell viability was assessed by performing the appropriate dilutions and plating the intracellular bacterial cells on PCA Viability was determined following 24 h incubation, at 37 °C. Infections were performed in quadruplicate with 3 biological replicates per experiment^58^.

### Statistical analysis

One-way ANOVA was performed for multivariant analysis followed by Tukey’s or Dunnett’s multiple comparison tests using GraphPad Prism V 6.0a.

## Additional Information

**How to cite this article**: Mina, E. G. and Marques, C. N. H. Interaction of *Staphylococcus aureus* persister cells with the host when in a persister state and following awakening. *Sci. Rep.* **6**, 31342; doi: 10.1038/srep31342 (2016).

## Supplementary Material

Supplementary Information

## Figures and Tables

**Table 1:** *S. aureus* relative fluorescence when infecting and colonizing *C. elegans* nematodes using the fast infection model.

Population	Relative Fluorescence x 10^7^		
	Day 1	Day 3	Day 5
Persister	0.4 ± 0.2	0.5 ± 0.03	2.2 ± 1.2
Persister + cis-DA	3.3 ± 1.8	1.7 ± 1.3	4.0 ± 3.5
Total	3.2 ± 1.9	2.0 ± 2.0	3.3 ± 2.0
Total + cis-DA	1.8 ± 1.3	1.2 ± 0.4	1.2 ± 0.8
Total 10^2^	3.4 ± 2.3	1.7 ± 1.2	2.4 ± 1.4
Total 10^2^ + cis-DA	1.5 ± 0.9	4.7 ± 2.7	1.0 ± 0.6

Epifluorescence microscopy was used to monitor the fluorescence distribution within the nematodes overtime.
