# Supplementary material for: Interaction of Staphylococcus aureus persister cells with the host when in a persister state and following awakening
Source: Sci Rep. 2016 Aug 10;6:31342. doi: 10.1038/srep31342 (PMC4979210; doi:10.1038/srep31342)
Supplement: Supplementary Information [file srep31342-s1.pdf]

Interaction of *Staphylococcus aureus* persister cells with the host when in a persister state and following awakening.

Mina, EG and Marques, CNH

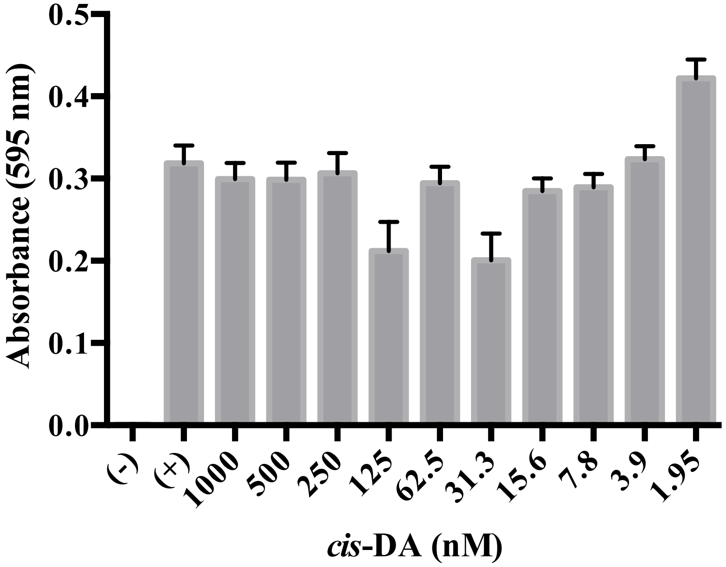

Fig. S1. Inhibition of *S. aureus* by the presence of *cis*-DA. Planktonic cultures of *S. aureus* were growth in LB containing different concentrations of *cis*-DA. Absorbance (595 nm) was read at time 0 and after 24 h of growth. (-) medium alone; (+) medium with *S. aureus*. Results are the average of 3 experiments with a total of at least 20 biological replicates. No significant difference was observed in *S. aureus* bacterial growth indicating that its growth is not inhibited in the presence of *cis*-DA at the concentrations tested.

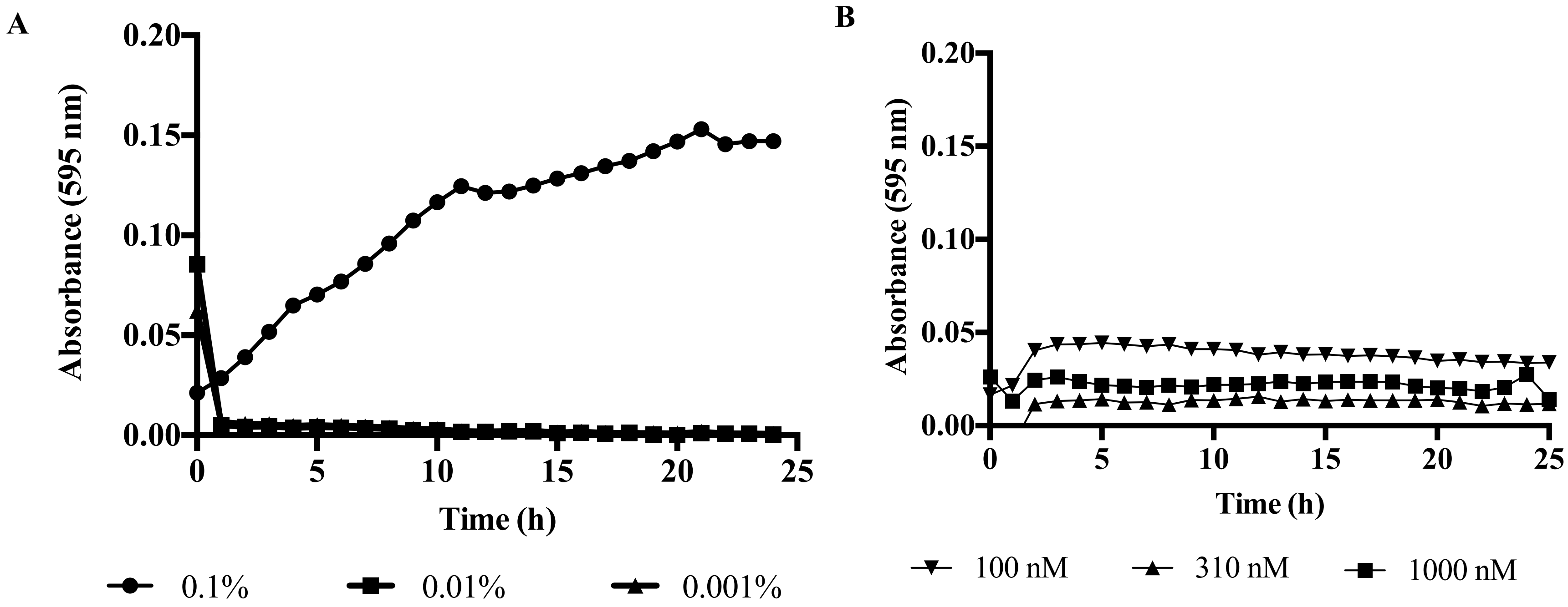

Fig. S2. Use of *cis*-DA as carbon source. Planktonic cultures of *S. aureus* were growth in EPRI containing, either peptone or *cis*-DA as a carbon source. Concentrations of peptone (A) consisted of 0.1%, 0.01% and 0.001%. Concentrations of *cis*-DA (B) consisted of 100 nM, 300 nM and 1000 nM. Results are the average of 3 experiments with a total of at least 20 biological replicates. Representative data is shown.
